# Genetic Variants of *HOTAIR* Associated With Colorectal Cancer Susceptibility and Mortality

**DOI:** 10.3389/fonc.2020.00072

**Published:** 2020-02-07

**Authors:** Jung Oh Kim, Hak Hoon Jun, Eo Jin Kim, Jeong Yong Lee, Han Sung Park, Chang Soo Ryu, Seungki Kim, Doyeun Oh, Jong Woo Kim, Nam Keun Kim

**Affiliations:** ^1^Department of Biomedical Science, College of Life Science, CHA University, Seongnam-si, South Korea; ^2^Department of Surgery, Bundang CHA Medical Center, School of Medicine, CHA University, Seongnam-si, South Korea; ^3^Department of Internal Medicine, Asan Medical Center, University of Ulsan College of Medicine, Seoul, South Korea; ^4^Department of Internal Medicine, Bundang CHA Medical Center, School of Medicine, CHA University, Seongnam-si, South Korea

**Keywords:** *HOTAIR*, long non-coding RNA, colorectal cancer, single nucleotide polymorphisms, case-control study

## Abstract

In colorectal carcinogenesis, the unique molecular and genetic changes that occur within cells result in specific CRC phenotypes. The involvement of the long non-coding RNA, *HOTAIR*, in cancer development, progression, and metastasis is well-established. Various studies have reported on the contribution of *HOTAIR* to cancer pathogenesis. Therefore, we selected four *HOTAIR* polymorphisms (rs7958904G>C, rs1899663G>T, rs4759314A>G, and rs920778T>C) to evaluate the association of each variant with CRC prevalence and prognosis. We conducted a case–control study of 850 individuals to identify the genotype frequencies of each polymorphism. The study population included 450 CRC patients and 400 control individuals that were randomly selected following a health screening. Notably, rs7958904 and rs1899663, their hetero genotype, and the dominant model were significantly different when compared to the healthy control group (rs7958904; AOR = 1.392, 95% CI = 1.052–1.843, *P* = 0.021). To evaluate the effect of *HOTAIR* polymorphisms on the survival rate, we analyzed patient mortality and relapse occurrence within 3 and 5 years with Cox-regression analysis. The rs7958904 CC polymorphism mortality rate was significantly higher than the GG polymorphism mortality rate (adjusted HR = 2.995, 95% CI = 1.189–7.542, *P* = 0.021). In addition, the rs920778 CC genotype was significantly different than the TT genotype (adjusted HR = 3.639, 95% CI = 1.435–9.230, *P* = 0.007). In addition, this study confirmed that genetic variants of *HOTAIR* alter the mRNA expression level (*P* < 0.01). We suggest that *HOTAIR* rs7958904G>C which is associated with CRC prevalence and mortality is a potential biomarker for CRC. The association between *HOTAIR* gene polymorphisms and CRC prevalence were reported for the first time.

## Introduction

Colorectal cancer (CRC) is the third most common type of cancer and the second leading cause of cancer-related mortalities in Western countries (1, 2). The prognosis of patients with CRC depends on the tumor stage at the time of diagnosis. Alarmingly, over 57% of patients already exhibit regional or distant tumor cell spreading when the cancer is first diagnosed (1). The pathogenesis of CRC usually follows a stepwise progression that begins with a benign polyp and advances to invasive adenocarcinoma. In colorectal carcinogenesis, the unique molecular and genetic changes that occur within cells characterize the specific CRC phenotype. CRC phenotypes are associated with variable tumor behaviors that are relevant to disease prognosis and therapeutic efficacy. It was also reported that many various factors had an effect on CRC susceptibility and prognosis. Accordingly, an active area of research within the field is the assessment of biomarkers that can be used to predict disease prognosis or the response to therapy, which will contribute to individualized disease management.

The involvement of *HOTAIR*, a long non-coding RNA (lncRNA) in cancer development, progression, and metastasis is well-known. The oncogenic lncRNA is involved in the progression of multiple human cancers including breast, gastric, pancreatic, liver, hepatocellular, colon, lung, colorectal, and ovarian cancer. *HOTAIR* levels are also a predictive cancer biomarker, and *HOTAIR* expression is highly correlated with patient prognosis. *HOTAIR* promotes different processes including tumor growth, metastasis, invasion and migration, the epithelial-to mesenchymal-transition (EMT), and stemness via cancer-type specific pathways. These cancer phenotypes predominantly occur through *HOTAIR*-mediated epigenetic changes, which illustrates that lncRNA-guided mechanisms can be hijacked in the context of cancer (3–6).

Various studies have reported on the contribution of *HOTAIR* to cancer pathogenesis. A gene silencing mechanism that is activated by histone protein modification is caused by *HOTAIR* overexpression. *HOTAIR* is also directly involved in the translational regulation of target mRNA. It has also been suggested that *HOTAIR* functions as a molecular decoy in tumors where it sequesters several microRNAs (miRNAs) and RNA binding proteins (RBPs). For example, in esophageal and epithelial cancer, *HOTAIR* acts as a competing endogenous RNA to negatively regulate miR-148a. Suppression of miR-148a promotes the expression of Snail2, which is a key driver of the EMT in cancer, to enhance cell invasion and metastasis (7, 8). Interestingly, a subset of miRNAs, including miR-141, can regulate *HOTAIR* expression by targeting *HOTAIR* to the RNA-induced silencing complex for subsequent degradation by Ago2-induced cleavage (7, 9). In addition, HOTAIR can accelerate colon cancer development by down-regulating miRNA-34a (10). In renal carcinoma, *HOTAIR* can bind with the RBP human antigen R, which then directs *HOTAIR* to the Let7 miRNA-Ago2 complex and leads to microRNA-mediated suppression of *HOTAIR* through degradation (11, 12). Notably, proteins or miRNAs that bind to *HOTAIR* can repress its function, which adds another layer of complexity to the regulation of *HOTAIR* activity.

Several studies (13–15) report that *HOTAIR* is upregulated in esophageal squamous-cell carcinoma (ESCC), and that elevated *HOTAIR* expression is related to advanced TNM stage and poor histological differentiation. Molecular studies (16) revealed that *HOTAIR* combined with polycomb repressive complex 2 (PRC2) promoted histone H3K27 methylation of the define (WIF1) promoter, which reduced the WIF1 protein synthesis and expression. Consequently, the reduced ß-catenin degradation and the increased T-cell factor/lymphoid enhancer-binding factor levels activated the Wnt/β-catenin signaling pathway (17). This process eventually increased the expression of target genes and enhanced tumor cell proliferation, invasion, and metastasis. *HOTAIR* may also promote EMT. *HOTAIR* silencing increases E-cadherin expression and is accompanied by a decreased vimentin and differential matrix metalloproteinase (MMP) nine expression in colon cancer cells. Collectively, these downstream events suggest that *HOTAIR* may function as a novel pleiotropic regulator of the EMT (18). Furthermore, Xu et al. reported that *HOTAIR* silencing in gastric cancer cells downregulated MMP1 and MMP3 expression and inhibited invasion; whereas upregulation of E-cadherin and differential zonula occludens-1 expression reversed the EMT (7). *HOTAIR* can be influenced by miRNA regulation and EMT promotion in carcinogenesis, cancer development, and metastasis suggested by many previous studies. Factors that can regulate the expression of HOTAIR can provide new clues to cancer treatment and coping.

Recent studies demonstrate an association between *HOTAIR* polymorphisms and CRC (19, 20). Recent studies suggested that SNPs of HOTAIR (such as rs920778, rs4759314, rs1899663, rs12826786, rs874945, rs7958904, and rs10783618) acted as potential cancer susceptibility loci and were significantly associated with the increased risk of various cancers. However, there are no studies on the association of these five SNPs in Koreans. Therefore, we selected four *HOTAIR* polymorphisms (rs7958904G>C, rs1899663G>T, rs4759314A>G, and rs920778T>C) to evaluate the association between HOTAIR variants and CRC prevalence and prognosis. The minor allele frequencies of the selected polymorphisms are >5% in the Korean population.

## Materials and Methods

### Ethics Statement

All study protocols of participants were reviewed and approved by The Institutional Review Board of CHA Bundang Medical Center and followed the recommendations of the Declaration of Helsinki. Study subjects were recruited from the South Korean provinces of Seoul and Gyeonggi-do between 1996 and 2009. The Institutional Review Board of CHA Bundang Medical Center approved this genetic study in June 2009 (IRB No. 2009-08-077) and informed consent was obtained from study participants.

### Study Population

We conducted a case–control study of 890 individuals. Four hundred and fifty patients that were diagnosed with CRC at the CHA Bundang Medical Center (Seongnam, South Korea) were enrolled from June 1996 to January 2009. The study only included CRC patients who had undergone surgical resection with a curative intent and who had histologically-proven adenocarcinoma. Collectively, there were 272 consecutive patients with colon cancer, 189 consecutive patients with rectal cancer, and 13 consecutive patients with unclassified CRC that underwent primary surgery. We retrospectively obtained patient information including the diagnosis date, the pathological stage, relapse events, and survival. Tumor staging of CRCs was performed according to the Sixth Edition of the American Joint Committee on Cancer (AJCC) staging manual. The control group was comprised of 416 individuals that were randomly selected following a health screening. This screening excluded patients with a history of thrombotic diseases or cancer. Patients with a high baseline blood pressure (systolic ≥140 mmHg or diastolic ≥90 mmHg) on more than one occasion or a history of antihypertensive medication were classified as having hypertension (HTN). Patients with a high fasting plasma glucose level (≥126 mg/dL), individuals who took oral hypoglycemic agents, or those with a history of insulin treatment were classified as having diabetes mellitus (DM). All study subjects were of Korean ethnicity and provided written informed consent. The study protocol was approved by the Institutional Review Board of the CHA Bundang Medical Center, Seongnam, South Korea.

### Phenotype Measurements

Anthropometric measurements, including body mass index (BMI), were collected for each individual. Systolic and diastolic blood pressures were measured in the seated position after 10 min of rest. To measure physiological parameters, 3 mL of blood were collected after overnight fasting. Plasma glucose levels were measured in duplicate by adapting the hexokinase method for use with an automated analyzer (TBA 200FR NEO, Toshiba Medical Systems, Tokyo, Japan). High density lipoprotein-cholesterol (HDL-C) levels were measured with enzymatic colorimetric methods using commercial reagent sets (TBA 200FR NEO, Toshiba Medical Systems). The homocysteine (Hcy) plasma concentration was measured with a fluorescent polarizing immunoassay (FPIA) with IMx (Abbott Laboratories, Chicago, IL, USA). The folate (FA) plasma concentration was measured with a radioimmunoassay kit (ACS:180; Bayer, Tarrytown, NY, USA).

### Genotyping

DNA was extracted from leukocytes using a G-DEX II Genomic DNA Extraction kit (Intron Biotechnology, Seongnam, Korea) according to the manufacturer's directions. To analyze *HOTAIR* genotypes, polymerase chain reaction-restriction fragment length polymorphism (PCR-RFLP) and TaqMan allele discrimination analysis were chosen because these procedures were more economical and cost-effective when compared to entire gene sequencing. Detailed conditions for PCR-RFLP, real-time PCR, and qRT-PCR methods are presented in Supplementary Materials. To validate the RFLP findings for each polymorphism, 30% of the PCR assays were randomly selected and duplicated, and followed by DNA sequencing. Sequencing was performed with an ABI 3730xl DNA Analyzer (Applied Biosystems, Foster City, CA, USA). The concordance of the quality control samples was 100%.

### Statistical Analysis

To analyze baseline characteristics of CRC and control patients, we used chi-square tests for categorical data and Student's *t*-tests for continuous data (21, 22). We estimated the association of VEGF and KDR polymorphisms with colorectal cancer incidence using adjusted odds ratios (AORs) and 95% confidence intervals (95% CIs) with multivariate logistic regression that was adjusted for age, gender, HTN, DM, BMI, and HDL-C. We chose HTN, DM, BMI, and HDL-C as adjustment variables because the risk factors for metabolic syndrome are closely associated with colorectal cancer. Cox-regression models were used to analyze the independent prognostic importance of various markers, and the results, which excluded 100 CRC patients that had insufficient medical history, were adjusted for age, gender, tumor differentiation, tumor site, chemotherapy, and cancer stage. Overall survival (OS) was defined as the time period from surgery until death or the last follow-up. Relapse-free survival (RFS) was defined as the time period from surgery until cancer reoccurred or the last follow-up. Hazard ratios (HRs) are presented with a 95% CI. Participants were followed for a median of 34 months (range, 4–173 months). The estimated 3-years OS and RFS rates for all patients were 82.6 and 81.7%, respectively. Analyses were performed using GraphPad Prism 4.0 (GraphPad Software Inc., San Diego, CA, USA) and Medcalc Version 12.7.1.0 (Medcalc Software, Mariakerke, Belgium). Haplotypes for multiple loci were estimated using the expectation-maximization algorithm with SNPAlyze (Version 5.1; DYNACOM Co, Ltd, Yokohama, Japan).

## Results

We evaluated *HOTAIR* gene polymorphisms (rs7958904, rs1899663, rs4759314, and rs920778) in CRC patients. We first classified the characteristics of patients in each polymorphism group, which included dividing the CRC patients into two groups based on the cancer location. Healthy control and colorectal patients were also age and gender matched. The detail information for sex ratios between the control and CRC patients were 41.6 and 45.1%, respectively, and the mean age was 61.2 and 62.1 years. There was no statistical significance between the two groups. The hypertension proportion in participants was higher, 40.4%, in the control group, compared with 33.1% in CRC patients (*P* = 0.030). Also, the total subject ratio of patients with diabetes mellitus was 13.2% in the control group and 16.0% of CRC patients showed a similar frequency between the two groups. In comparing the mean levels of plasma folate and plasma homocysteine, folate was found to be 9.0 in the control group and 7.8 in the patient group (*P* < 0.0001). However, homocysteine did not show any difference between the two groups (Table 1).

**Table 1:** Baseline characteristics between CRC patients and healthy control subjects.

**Characteristic**	**Control (*n* = 416)**	**CRC (*n* = 474)**	P^*^	**Colon (*n* = 272)**	P^*^	**Rectum (*n* = 189)**	P^*^
Male, *n* (%)	173 (41.6)	214 (45.1)	0.317	122 (44.9)	0.443	84 (44.4)	0.568
Age (mean ± SD)	61.2 ± 11.38	62.1 ± 12.48	0.230	61.9 ± 12.97	0.267	62.1 ± 11.81	0.344
HTN, n (%)	168 (40.4)	157 (33.1)	0.030	86 (31.6)	0.025	68 (36)	0.347
DM, n (%)	55 (13.2)	76 (16.0)	0.277	46 (16.9)	0.220	30 (15.9)	<0.0001
Folate (mean ± SD)	9.0 ± 8.05	7.8 ± 6.82	<0.0001	7.9 ± 6.77	<0.0001	7.7 ± 6.97	<0.0001
Hcy (mean ± SD)	9.8 ± 4.25	10.7 ± 7.8	0.330	10.4 ± 8.12	0.870	10.9 ± 7.34	0.096
TNM stage							
I		45 (9.5)		20 (7.4)		24 (12.7)	
II		188 (39.7)		115 (42.3)		70 (37)	
III		189 (39.9)		109 (40.1)		78 (41.3)	
IV		46 (9.7)		27 (9.9)		16 (8.5)	
Tumor size							
T < 5 cm		275 (58)		176 (64.7)		96 (50.8)	
T ≥ 5 cm		187 (39.5)		92 (33.8)		93 (49.2)	

*CRC, colorectal cancer; SD, standard deviation; HTN, hypertension; DM, diabetes mellitus; Hcy, plasma homocysteine; TNM, tumor node metastasis*.

* *P-values were calculated using chi-squared tests for categorical data and two–sided t-tests for continuous data*.

In Table 2, we identified the genotype frequencies of each polymorphism. The rs7958904 and rs1899663 polymorphisms, their hetero genotypes, and dominant models were significantly different when compared to the healthy control group (*HOTAIR* rs7958904 GG vs. GC: AOR = 1.352, 95% CI = 1.014–1.801, *P* = 0.040; GG vs. GC+CC: AOR = 1.351, 95% CI = 1.027–1.777, *P* = 0.032; *HOTAIR* rs1899663 GG vs. GT: AOR = 1.3385, 95% CI = 1.004–1.784, *P* = 0.047; GG vs. GT+TT: AOR = 1.378, 95% CI = 1.043–1.822, *P* = 0.024). However, the rs4759314 and rs920778 polymorphisms were not statistically different when compared with the control group. Additionally, rectal cancer patients (189 samples) exhibited a significant difference with respect to both the rs7958904 GC type polymorphism (AOR = 1.559, 95% CI = 1.076–2.258, *P* = 0.019) and the dominant model (AOR = 1.547, 95% CI = 1.085–2.205, *P* = 0.016) when compared with the control patients. Although the rs1899663 GT type polymorphism was not statistically significant, the dominant model was statistically different when compared to control patients (AOR = 1.481, 95% CI = 1.035–2.120, *P* = 0.032). Interestingly, patients with localized colon cancer did not exhibit any differences when compared with healthy controls (*P* > 0.05).

**Table 2:** Comparison of *HOTAIR* polymorphism genotype frequencies between CRC patients and healthy controls.

**Genotypes**	**Controls (*n* = 416)**	**CRC (*n* = 474)**	**AOR (95% CI)^a^**	***P^a^***	**Colon (*n* = 272)**	**AOR (95% CI)^a^**	***P^a^***	**Rectum (*n* = 189)**	**AOR (95% CI)^a^**	***P^a^***
***HOTAIR*** **rs7958904 G>C**										
GG	249 (59.9)	244 (51.5)	1.000 (reference)		151 (55.5)	1.000 (reference)		89 (47.1)	1.000 (reference)	
GC	140 (33.7)	191 (40.3)	1.352 (1.014–1.801)	0.040	102 (37.5)	1.202 (0.859–1.681)	0.283	84 (44.4)	1.559 (1.076–2.258)	0.019
CC	27 (6.5)	39 (8.2)	1.399 (0.802–2.443)	0.237	19 (7)	1.364 (0.7–2.66)	0.362	16 (8.5)	1.516 (0.751–3.062)	0.246
Dominant			1.351 (1.027–1.777)	0.032		1.206 (0.875–1.663)	0.252		1.547 (1.085–2.205)	0.016
Recessive			1.168 (0.681–2.002)	0.573		1.144 (0.602–2.175)	0.682		1.235 (0.626–2.435)	0.543
HWE-*P*	0.230	0.850			0.755			0.538		
***HOTAIR*** **rs1899663 G>T**										
GG	271 (65.1)	264 (55.7)	1.000 (reference)		161 (59.2)	1.000 (reference)		101 (53.4)	1.000 (reference)	
GT	132 (31.7)	186 (39.2)	1.338 (1.004–1.784)	0.047	100 (36.8)	1.219 (0.871–1.707)	0.248	76 (40.2)	1.404 (0.968–2.038)	0.074
TT	13 (3.1)	24 (5.1)	1.783 (0.86–3.698)	0.120	11 (4)	1.608 (0.671–3.854)	0.287	12 (6.3)	2.141 (0.906–5.06)	0.083
Dominant			1.378 (1.043–1.822)	0.024		1.244 (0.897–1.725)	0.191		1.481 (1.035–2.12)	0.032
Recessive			1.559 (0.764–3.182)	0.223		1.387 (0.591–3.256)	0.452		1.958 (0.849–4.515)	0.115
HWE-*P*	0.523	0.228			0.350			0.646		
***HOTAIR*** **rs4759314 A>G**										
AA	358 (86.1)	395 (83.3)	1.000 (reference)		229 (84.2)	1.000 (reference)		159 (84.1)	1.000 (reference)	
AG	55 (13.2)	71 (15)	1.062 (0.717–1.574)	0.763	39 (14.3)	1.031 (0.651–1.632)	0.898	28 (14.8)	1.026 (0.611–1.721)	0.924
GG	3 (0.7)	8 (1.7)	1.017 (0.201–5.139)	0.984	4 (1.5)	0.622 (0.063–6.126)	0.684	2 (1.1)	1.722 (0.28–10.577)	0.557
Dominant			1.059 (0.721–1.557)	0.770		1.012 (0.644–1.591)	0.960		1.056 (0.639–1.746)	0.831
Recessive			0.994 (0.197–5.016)	0.994		0.62 (0.063–6.091)	0.682		1.658 (0.27–10.172)	0.585
HWE-*P*	0.581	0.027			0.130			0.545		
***HOTAIR*** **rs920778 T>C**										
TT	241 (57.9)	258 (54.4)	1.000 (reference)		149 (54.8)	1.000 (reference)		102 (54)	1.000 (reference)	
TC	149 (35.8)	180 (38)	1.115 (0.838–1.484)	0.457	103 (37.9)	1.152 (0.825–1.607)	0.407	73 (38.6)	1.075 (0.74–1.56)	0.706
CC	26 (6.3)	36 (7.6)	1.222 (0.699–2.135)	0.482	20 (7.4)	1.353 (0.704–2.599)	0.365	14 (7.4)	1.118 (0.54–2.317)	0.763
Dominant			1.124 (0.855–1.477)	0.402		1.167 (0.849–1.604)	0.342		1.076 (0.754–1.535)	0.688
Recessive			1.139 (0.661–1.962)	0.640		1.228 (0.653–2.307)	0.524		1.058 (0.519–2.159)	0.876
HWE-*P*	0.645	0.555			0.706			0.851		

*CRC, colorectal cancer; AOR, adjusted odds ratio; 95% CI, 95% confidence interval*.

a *Adjusted by age, gender, HTN, DM*.

**Figure 1:**
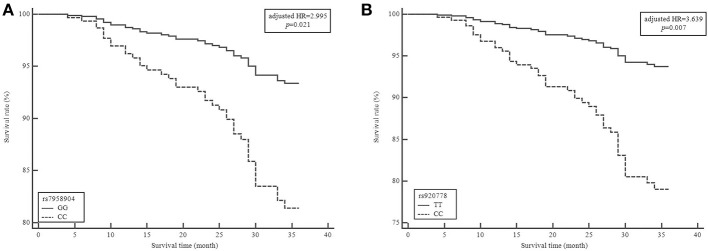
Survival plot from a Cox proportiona hazards model with HOTAIR rs7958904G>C and rs920778T>C polymorphisms in colorectal cancer (CRC). Survival curve of patients grouped by 3 years mortality in colorectal cancer based on **(A)** HORAIR rs7958904GG vs. HOTAIR rs7958904CC genotypes **(B)** HORAIR rs920778TT vs. HOTAIR rs920778CC genotypes. HR, hazard ratio.

To evaluate the effect *HOTAIR* polymorphisms on survival rate, we analyzed mortality and 3-years relapse occurrence with Cox-regression analysis. The *HOTAIR* rs7958904 CC genotype mortality was significantly higher than the GG genotype (adjusted HR = 2.995, 95 %CI = 1.189–7.542, *P* = 0.021). Additionally, the *HOTAIR* rs920778 CC genotype was significantly different when compared to the TT genotype (adjusted HR = 3.639, 95% CI = 1.435–9.230, *P* = 0.007; Table 3 and Figure 1). To complete the subgroup analysis, we analyzed the mortality of colon cancer patients, and found that patients with *HOTAIR* rs7958904 and rs920778 polymorphisms had greater survival rates. Interestingly, the *HOTAIR* rs1899663 TT genotype was associated with increased colon cancer mortality (adjusted HR = 4.507, 95% CI = 1.167–17.413, *P* = 0.030), and the association with increased mortality only held for colon cancer (Table 3).

**Table 3:** *HOTAIR* polymorphism genotype frequencies and CRC patient 3-years mortality in overall, colon, and rectum cancer.

**Genotypes**	**Total CRC (*n* = 474)**	**Death (*n* = 74)**	**Adjusted HR(95% CI)^†^**	***P***	**Colon (*n* = 272)**	**Death (*n* = 43)**	**Adjusted HR (95% CI)^†^**	***P***	**Rectum (*n* = 189)**	**Death (*n* = 29)**	**Adjusted HR (95% CI)^†^**	***P***
***HOTAIR*** **rs7958904 G>C**												
GG	244 (51.5)	40 (54.1)	1.000 (reference)		151 (55.5)	27 (62.8)	1.000 (reference)		89 (47.1)	11 (37.9)	1.000 (reference)	
GC	191 (40.3)	27 (36.5)	0.891 (0.501–1.584)	0.695	102 (37.5)	13 (30.2)	0.659 (0.299–1.452)	0.304	84 (44.4)	14 (48.3)	1.797 (0.704–4.582)	0.222
CC	39 (8.2)	7 (9.5)	2.995 (1.189–7.542)	0.021	19 (7)	3 (7)	4.835 (1.094–21.376)	0.039	16 (8.5)	4 (13.8)	6.076 (1.125–32.814)	0.037
Dominant			1.070 (0.633–1.810)	0.801			0.820 (0.401–1.675)	0.587			2.039 (0.850–4.892)	0.112
Recessive			2.583 (1.086–6.145)	0.033			3.208 (0.877–11.738)	0.080			3.383 (0.893–12.818)	0.075
***HOTAIR*** **rs1899663 G>T**												
GG	264 (55.7)	45 (60.8)	1.000 (reference)		161 (59.2)	29 (67.4)			101 (53.4)	15 (51.7)	1.000 (reference)	
GT	186 (39.2)	23 (31.1)	0.732 (0.401–1.336)	0.312	100 (36.8)	11 (25.6)	0.499 (0.216–1.152)	0.105	76 (40.2)	11 (37.9)	1.606 (0.619–4.171)	0.333
TT	24 (5.1)	6 (8.1)	2.328 (0.876–6.185)	0.092	11 (4)	3 (7)	4.507 (1.167–17.413)	0.030	12 (6.3)	3 (10.3)	7.110 (0.831–60.815)	0.075
Dominant			0.874 (0.508–1.503)	0.627			0.691 (0.331–1.443)	0.327			1.592 (0.668–3.792)	0.296
Recessive			2.227 (0.871–5.693)	0.096			4.468 (1.263–15.812)	0.021			1.576 (0.316–7.866)	0.581
***HOTAIR*** **rs4759314 A>G**												
AA	395 (83.3)	61 (82.4)	1.000 (reference)		229 (84.2)	37 (86)			159 (84.1)	23 (79.3)	1.000 (reference)	
AG	71 (15)	13 (17.6)	1.102 (0.555–2.188)	0.782	39 (14.3)	6 (14)	0.739 (0.276–1.979)	0.549	28 (14.8)	6 (20.7)	1.462 (0.471–4.539)	0.513
GG	8 (1.7)	0 (0)	N/A	0.991	4 (1.5)	0	N/A	0.991	2 (1.1)	0	N/A	0.990
Dominant			1.007 (0.507–1.997)	0.985			0.694 (0.262–1.840)	0.465			1.265 (0.406–3.938)	0.687
Recessive			N/A	0.967			N/A	0.967			N/A	0.990
***HOTAIR*** **rs920778 T>C**												
TT	258 (54.4)	44 (59.5)	1.000 (reference)		149 (54.8)	27 (62.8)			102 (54)	15 (51.7)	1.000 (reference)	
TC	180 (38)	22 (29.7)	0.754 (0.410–1.385)	0.365	103 (37.9)	12 (27.9)	0.683 (0.310–1.505)	0.347	73 (38.6)	10 (34.5)	1.208 (0.438–3.334)	0.717
CC	36 (7.6)	8 (10.8)	3.639 (1.435–9.230)	0.007	20 (7.4)	4 (9.3)	12.964 (2.216–75.858)	0.005	14 (7.4)	4 (13.8)	3.925 (0.860–17.908)	0.079
Dominant			0.985 (0.570–1.700)	0.956			0.901 (0.440–1.844)	0.776			1.493 (0.611–3.648)	0.382
Recessive			3.481 (1.456–8.324)	0.005			8.143 (1.946–34.084)	0.004			3.383 (0.893–12.818)	0.075

† *Adjusted for age, sex, hypertension, diabetes mellitus, tumor size, lymph node metastasis, chemotherapy, smoking, and alcohol based on Cox-regression analysis*.

**Figure 2:**
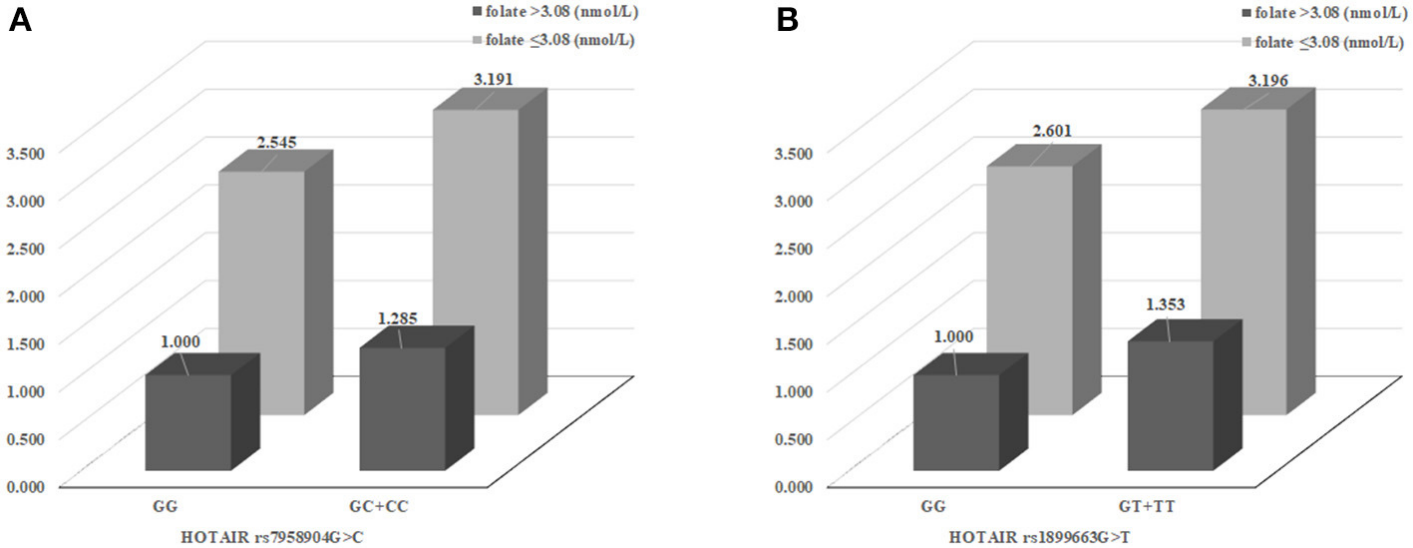
The effects of HOTAIR rs7958904 G>C and rs1899663 variant on colorectal cancer (CRC) risk modulated by clinical factors. **(A)** The synergic effect for CRC susceptibility combined between HOTAIR rs7958904 GC+CC and low plasma folate group (AOR, 3.191) than rs7958904 GC+CC with high plasma folate group (AOR, 1.285) or rs7958904 GG with low plasma folate group (AOR, 2.545). **(B)** The HOTAIR rs1899663 G>T were show to elevated CRC prevalence, in the case of *HOTAIR* rs1988663 GT+TT with low plasma folate group (AOR, 3.196) than rs1988663 GT+TT with high plasma folate group (AOR, 1.353) or rs1899663 GG with low plasma folate group (AOR, 2.601).

We evaluated synergic effect between clinical parameters and *HOTAIR* polymorphisms (Supplementary Table 1). In order to confirm the synergistic effects of clinical factors and genotypes, we performed stratified analysis based on the classification of sex, age, hypertension, diabetes mellitus, plasma folate, and homocysteine levels analyzed the interactions with *HOTAIR* polymorphisms. Especially, when analyzing the synergistic effect of increasing the risk of CRC with genotypes, according to the concentration of folate in plasma, it was found that the association of CRC risk was increased at low folate concentrations (≤ 3.08 nmol/L) group in *HOTAIR* rs7958904 GC+CC and rs1899663 GT+TT types (Figure 2).

We measured *HOTAIR* expression levels in colon cancer and normal tissues to determine the expression pattern of specific *HOTAIR* polymorphisms. The overall expression of *HOTAIR* was 2-fold greater in CRC when compared to control tissues (Supplementary Figure 1, *P* < 0.01). Next, to identify the altered expression patterns of the specific *HOTAIR* genotype, rs7958904G>C, we analyzed the expression of the *HOTAIR* rs7958904G>C polymorphism compared with normal tissue and tumor tissue. The *HOTAIR* rs7958904 CC genotype was highly expressed in tumor tissue than normal tissues (*P* < 0.01). However, GG and C genotypes do not have significantly different expression levels compared with normal tissue and tumor tissue (Supplementary Figure 2).

LD block and haplotype analyses were performed to identify linkage disequilibrium between SNPs. When analyzed using Haploview software (https://www.broadinstitute.org/haploview/haploview), it was confirmed that the *R*^2^ value was the highest at 0.810 between rs7958904 and rs1899663 in the analysis between four SNPs, but no LD block was generated (Supplementary Figure 3). The haplotype analysis (Supplementary Tables 2–4) revealed that the various haplotypes were at a higher risk. The data suggests that the C-T (*HOTAIR* rs7958904G>C/rs1899663G>T) haplotype is more prevalent in CRC when compared to the baseline (OR = 1.584, 95% CI = 1.117–2.247, *P* = 0.010). The C-G-A (*HOTAIR* rs7958904G>C/rs1899663G>T/rs4759314A>G) haplotype analysis revealed that the C-G-A haplotype was specifically associated with reduced CRC occurrence (OR = 0.212, 95%CI = 0.060–0.750, *P* = 0.008). Furthermore, we identified genotype combinations (Supplementary Tables 5, 6) where the *HOTAIR* rs1899663 GT genotype, when combined with other polymorphisms, had a synergistic effect that increased CRC risk. However, several genotype combinations demonstrated that the occurrence of cancer was location-dependent. The GC+GT (*HOTAIR* rs7598904+rs1899663) and AG+TT (*HOTAIR* rs4759314+rs920778) genotypes were significantly different in rectal cancer patients, but there was not any statistical significance in colon cancer patients. Supplementary Table 5 summarizes the statistical powers of positive genetic associations in the present study.

## Discussion

In this study, we analyzed the association between the risk development of four SNPs of HOTAIR in Korean CRC patients and the correlation between CRC mortality by survival analysis. In addition, we confirmed that the HOTAIR expression for rs7958904G> C showed the strongest statistical correlation. Interestingly, our results confirm the association between rs7958904G> C and rs1899663G> T rather than the previously known rs4759314A> G that is related to CRC susceptibility. It was confirmed that similar correlations were found in the colon and rectum regions.

The lncRNAs participate in gene regulation (23, 24). Numerous lncRNAs have been shown to promote cell invasion and metastasis (25, 26). *HOTAIR* is an lncRNA located in the HOXC locus, and it can interact with PRC2. This interaction mediates the methylation of lysine 27 and the demethylation of lysine 4 on histone H3 in the HOXD locus, where enhancer of zeste homolog 2 also plays a regulatory role (27, 28). *HOTAIR* can alter the state of chromosomes, which affects gene expression. Importantly, *HOTAIR* expression was upregulated in cancer tissue samples from patients with breast, pancreatic, liver, gastric, and non-small cell lung cancers, and elevated *HOTAIR* expression was even more pronounced in metastatic tissue. Both *in vivo* and *in vitro* studies confirm that upregulated expression of *HOTAIR* enhances tumors invasion and metastasis (28–30). For example, prior studies report that *HOTAIR* is significantly upregulated and closely related to invasion and metastasis in endothelial cells (15, 31). Studies with CRC patients revealed that *HOTAIR* expression levels are higher in CRC tissue when compared with corresponding non-cancerous tissue. Similar findings were reported for ovarian (32) and laryngeal squamous-cell cancers. High expression levels of *HOTAIR* are correlated with the presence of liver metastases, and CRC patients with elevated *HOTAIR* expression have a worse prognosis than patients with tumors exhibiting low *HOTAIR* expression (33). Based on an *in-situ* hybridization assay, 91 of 160 (56.87%) paraffin embedded nasopharyngeal carcinoma (NPC) biopsy specimens had elevated *HOTAIR* levels. NPC patients with high tumor *HOTAIR* expression levels had a worse OS prognosis than patients with low *HOTAIR* expression (34). Furthermore, elevated *HOTAIR* expression was detected in patients that had high histological grade tumors or advanced clinical stage cancer (35). Depletion of *HOTAIR* by short interfering RNA, decreased invasion and increased the apoptosis of human epithelial type 2 cells. In addition, tumor growth was significantly inhibited in mice injected with *HOTAIR*-deficient cells (35). Overexpression of *HOTAIR* was also strongly associated with high-grade tumors and metastasis in gastrointestinal stromal tumor specimens (36). ESCC patients with elevated levels of *HOTAIR* had significantly lower 5-years survival rates than *HOTAIR*-negative patients (15). Finally, high *HOTAIR* levels in primary sarcoma correlated with a high metastasis probability (37).

Recent studies identified that lncRNAs bind to miRNAs to “communicate” with other RNA targets (38). Interestingly, lncRNAs and miRNA exhibit reciprocal regulation. As many lncRNAs control important physiological functions, the abundance and binding of each miRNA and lncRNA directly alters cell function. By sharing common miRNA binding sites with mRNA targets, lncRNAs sequester and compete with miRNAs to inhibit miRNA functionality and alleviate mRNA repression (39). Interestingly, 40% of miRNAs are found in the introns of protein coding genes (40). Furthermore, analyses indicate that 10% of lncRNA genes also host an miRNA, either in an intron or exon (41). In addition, miRNAs bind to lncRNAs, and when combined with other RBPs, regulate lncRNA stability and miRNA-mediated decay. Recently, it was reported that RNA interference-mediated knockdown of *HOTAIR* altered the expression of *HOTAIR* target genes and suppressed the invasion of gastrointestinal stromal tumor cells. Another study revealed that *HOTAIR* functions as a “miRNA sponge” that silenced miRNAs (tumor suppressor), and thereby induced the overexpression of oncogenic genes (8).

At this point, HOTAIR expression has been well-studied. The mechanisms of cancer onset and metastasis, and HOTAIR SNP have also actively been studied. However, there are insufficient studies to establish the link between HOTAIR polymorphism and CRC, and some target SNPs of the previously reported HOTAIR polymorphism are limited. In particular, according to genotypes that vary by SNP, there are no existing reported studies on the changes in HOTAIR expression extending from the linkage study between SNP and CRC. Functional studies through studies on statistically related polymorphisms may be a way to confirm the contribution of HOTAIR regulation in cancer onset/metastasis and maybe a major guideline for cancer onset and prognosis. In our results, the C allele of rs7958904G>C was found to be the association for CRC susceptibility, mortality, and altered expression level. In previous studies, rs7958904 is a SNP present in 3′UTR of HOTAIR and it has been reported that SNPs located in 3′UTR. Regulation mechanisms of gene expression including mRNA stability, miRNA binding, cis-regulation. However, in some cases, the results of the analysis regarding the association of SNPs is not clear. In our results, there was not an LD block between the four SNPs we selected in the haplotype analysis, and the allele combination analysis showed that the CRC risk increased in some cases, even with the combination of minor alleles had shown to reduce the risk. This result is considered to be a variation that could have been caused by classifying the haplotype with a small sample size- Performing statistical progress based on these results, it is necessary to collect additional samples and analyze the population with more people.

CRC is associated with a variety of factors. Representative factors include hypertension (42, 43), folate (44, 45), homocysteine (46), and diabetes milieus (47). Thus, treatment strategies and prognostic management methods change. Therefore, it is well-known to check the association with clinical parameters, and it is necessary to perform disease management based on this. In addition, due to the development of many studies and techniques, the pathogenesis and diagnosis methods of CRC are developing, and the rate of CRC treatment by early diagnosis is increasing (25, 26, 48, 49). However, despite advances in technology, people in blind spots, those with the burden of medical expenses and the difficulties of living conditions do not receive these benefits (50–52). Therefore, there is an urgent need to find new therapeutic targets. In this regard, it is necessary to study markers for early diagnosis and treatment, and the HOTAIR polymorphism proposed in our study is expected to satisfy this need.

There are some limitations to our research. First, case-control studies were conducted only in the Korean population. However, the frequency of genotypes was consistent with studies in other populations previously reported and confirmed the frequency of meeting the HWE *p*-value. Although the size of our research sample was small, statistical verification was confirmed through statistical power analysis. Secondly, the duration of survival analysis in cancer research is generally 5 years, but our analysis is 3 years. This was done because when the data was compiled for a 5-years survival analysis, the explanatory power of the analysis decreased because there were too many samples that lost the survival information. Finally, although our study suggested a link between the HOTAIR SNP risk of CRC and survival analysis, it confirmed differences in HOTAIR expression for the genotype of rs7958904, RNA second structure changes, RNA stability regulation, and RNA of these SNPs. The mechanism of expression regulation such as the role of binding protein sites, was not elucidated.

In conclusion, this study analyzed the expression levels of *HOTAIR* and miRNAs in genetic variants. In addition, the association between *HOTAIR* gene polymorphisms and colorectal cancer prevalence, including the altered expression patterns of *HOTAIR* polymorphisms, were reported for the first time.

## Data Availability Statement

The raw data supporting the conclusions of this article will be made available by the authors, without undue reservation, to any qualified researcher.

## Ethics Statement

All study protocols of participants were reviewed and approved by The Institutional Review Board of HA Bundang Medical Center and followed the recommendations of the Declaration of Helsinki. Study subjects were recruited from the South Korean provinces of Seoul and Gyeonggi-do between 1996 and 2009. The Institutional Review Board of CHA Bundang Medical Center approved this genetic study in June 2009 (IRB No. 2009-08-077) and informed consent was obtained from study participants.

## Author Contributions

NK and JWK: onceptualization. JOK, EK, and JL: methodology. HP, CR, SK, and DO: validation. JOK, HJ, JL, HP, and CR: formal analysis. EK and SK: investigation. JWK and DO: resources. NK: data curation. JOK, HJ, and EK: writing—original draft preparation. JOK: writing—review and editing. NK and JWK: project administration.

### Conflict of Interest

The authors declare that the research was conducted in the absence of any commercial or financial relationships that could be construed as a potential conflict of interest.
