# Supplementary material for: Genetic Variants of HOTAIR Associated With Colorectal Cancer Susceptibility and Mortality
Source: Front Oncol. 2020 Feb 7;10:72. doi: 10.3389/fonc.2020.00072 (PMC7020018; doi:10.3389/fonc.2020.00072)
Supplement: Supplementary file 1 [file Table_1.DOCX]

Supplementary Material

# Supplementary Data

## PCR-RFLP & real-time PCR

The *HOTAIR* rs1899663 G>T was detected using a specific forward (5'-TTT TCC AGT TGA GGA GGG TGG A-3') and reverse primer (5'-CTA ATG GCA AGG GAA GGG AAG G-3'). The 114-bp PCR product was then digested with 5U of *Hph* I. A 114-bp digestion product represented the TT genotype; 114-bp, 79-bp, and 35-bp fragments represented the GT genotype; and 35-bp fragments represented the GG genotype.

The *HOTAIR* rs4759314 A>G polymorphism was also detected by PCR-RFLP analysis using specific forward (5'-ACC CAA AAC CAT TTC CTG AGA G-3') and reverse (5'-TTC AGG TTT TAT TAA CTT GCA TCA GC-3') primers. The 124-bp product was digested with 5U of *Alu* I for 16 h at 55°C. A restriction digest fragment of 124-bp represented the GG genotype; 124-bp, 99-bp, and 25-bp fragments represented the GA genotype; and 98-bp and 25-bp fragments represented the AA genotype.

To detect the *HOTAIR* rs920778 T>C genotypes, PCR-RFLP analysis was performed with specific forward (5'-GCC TCT GGA TCT GAG AAA GAA A-3') and reverse (5'-TTA CAG CTT AAA TGT CTG AAT GTT CC-3') primers. The length of the amplified fragment was 140 bp. PCR products were digested with 5U of *Nsp* I for 16 h at 37°C. For *HOTAIR* rs920778 T>C, restriction digest products that were 140 bp represented the TT genotype; 140-bp, 113-bp, and 27-bp fragments represented the TC genotype; and 113-bp and 27-bp fragments represented the CC genotype.

All PCR experiments were performed using an AccuPowerHotStart PCR PreMix (Bioneer Corporation, Daejeon, Korea). Polymorphisms were identified by digesting the rs920778 product with *Msp* I, the rs1899663 product with *Hph* I, and the rs4759314 PCR products with *Alu* I (New England BioLabs, Inc., Ipswich, MA, USA). These restriction digests were performed at 37°C for 16 h and detected using gel electrophoresis with a 4% agarose gel followed by visualization with ethidium bromide on a Gel Doc XR+ version system (Bio Rad, Hercules, CA, USA).

*HOTAIR* rs7958904 G>C polymorphism genotyping was determined using real-time PCR (RG-6000, Corbett Research, Australia) for allelic discrimination. Primer and TaqMan probes were designed using Primer Express Software (version 2.0; Thermo Fisher Scientific, Inc., Waltham, MA, USA). Primers were synthesized and supplied by Applied Biosystems (Foster City, CA, USA). The reporter dyes used were 5-carboxyfluorescein (FAM) and 2', 7'-dimethoxy-4', 5'-dichloro-6-carboxyfluorescein (JOE). For rs7958904, the selected probes were 5’-[JOE]-CG GCT CGG GTC AG-[BHQ1]-3’ (C allele detecting probe) and 5-’[FAM]-CG GCT CCG GTC AG-[BHQ1]-3’ (G allele detecting probe).

## Quantitative real-time PCR

To perform qRT-PCR, total RNA was extracted from 18 patient colorectal tissues (9 normal and 9 tumor tissues) using TRIzol reagent (Invitrogen, Grand Island, NY, USA) according to the manufacturer's instructions. Synthesis of cDNA from total RNA was completed with the SuperScript III First-Strand Synthesis System (Invitrogen, Grand Island, NY, USA). *HOTAIR* mRNA level were measured with qRT-PCR (RG-6000, Corbett Research, Australia). The *HOTAIR* mRNA expression levels in the 18 tissues were then compared by a comparative CT (2-ΔΔCT) method with glyceraldehyde 3-phosphate dehydrogenase (GAPDH) as internal housekeeping controls. The primer sequences for amplification were as follows: HOTAIR: forward 5’-CAG TGG GGA ACT CTG ACT CG-3’ and reverse 5’-GTG CCT GGT GCT CTC TTA CC-3’. GAPDH: forward 5’-GTC AAC GGA TTT GGT CTG TAT T-3’ and reverse 5’-AGT CTT CTG GGT GGC AGT GAT-3’. Each of the RNA samples was prepared with a reaction volume of 20 μl. The HB miR Multi Assay Kit System I (HeimBiotek, Korea) was used to analyze the miRNA in a two-step process. In the initial step, synthesis of complementary DNA was completed with the cDNA-HB I Reverse Transcription (RT) Reaction Kit, and the reagents were used according to the manufacturer's instructions. Then, amplification with reverse transcription PCR was completed with the following thermocycler (Eppendorf, Hamburg, Germany) conditions: Incubation at 37°C for 60 min (Step 1), incubation at 95°C for 5 min (Step 2), and an infinite hold at 4°C. This process yielded 20 μl of a final cDNA product for each respective total RNA sample. The final product was stored at ˗20°C for further use.

# Supplementary Figures and Tables

## Supplementary Figures


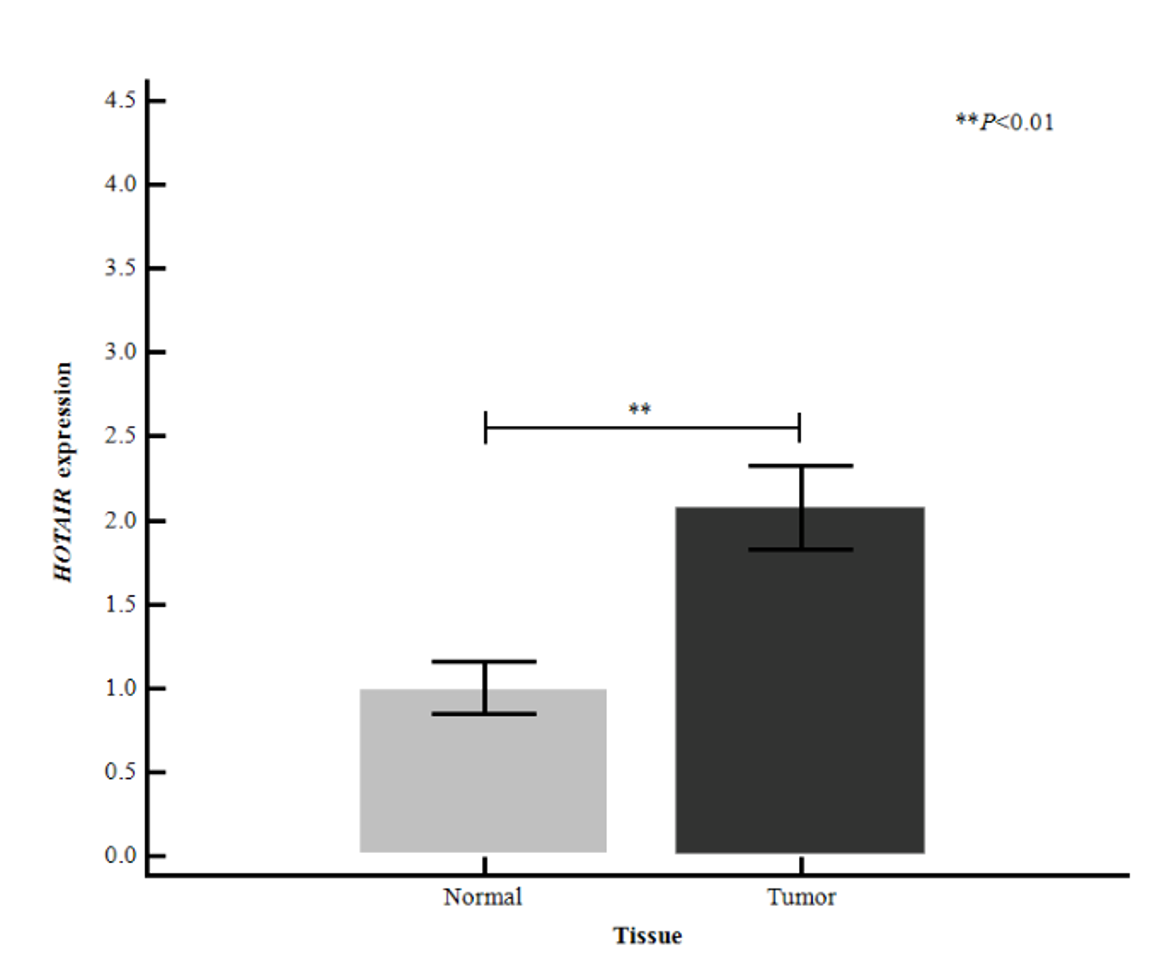


**Supplementary Figure 1. Altered expression level of HOTAIR in normal and colorectal cancer tissue.** Compared between normal and tumor tissue for the expression level of HOTAIR. The expression of HOTAIR appears to be about 2-fold change in tumor tissue compared to normal tissue, and there is a statistical significance (*P*<0.01).


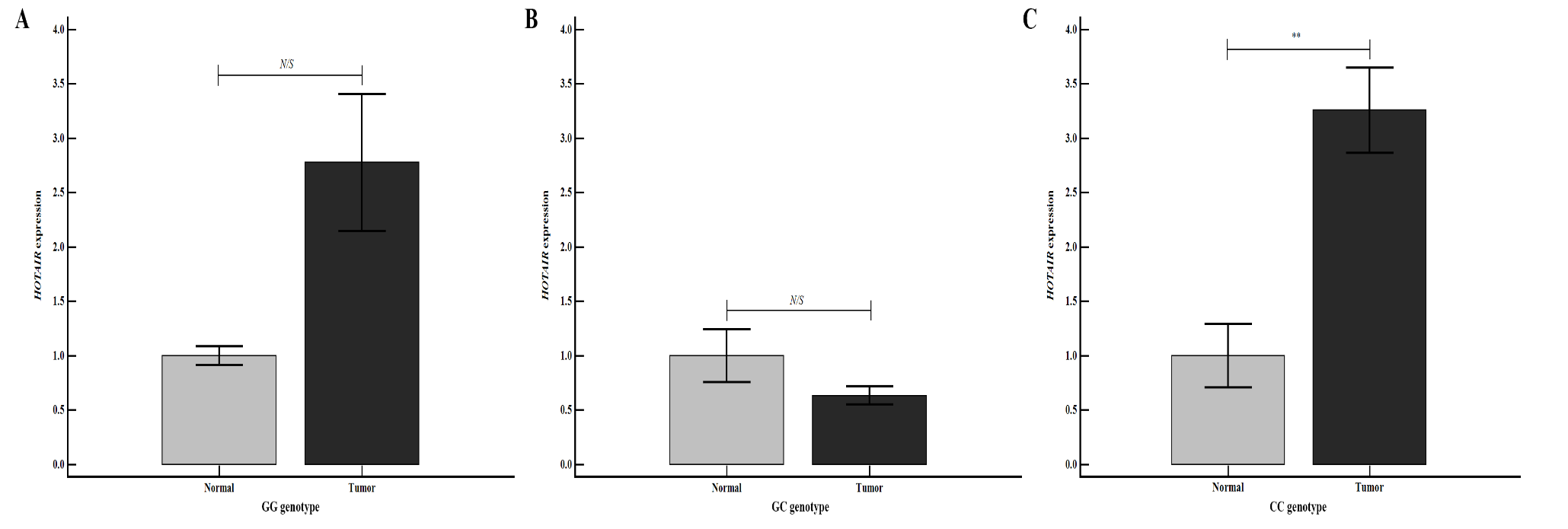


**Supplementary Figure 2. Difference expression level by HOTAIR rs7958904 genotype on normal and tumor tissue.** (A) When HOTAIR rs7958904 CC genotype, the expression of HOTAIR is increased markedly in tumor tissue than normal tissue and statistical significance presented (*P*<0.01). However, (B) GG and (C) GC genotypes, which have not significantly different expression levels compared to normal tissue and tumor tissue. Note; N/S, not significance; ** *P*<0.01.


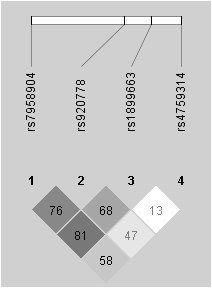


**Supplementary Figure 3. Linkage disequilibrium plots for four SNPs of the *HOTAIR* gene using Haploview software.** Based on the D ' value calculated by performing the linkage disequilibrium between the SNPs in the LD plot. Pairwise LD calculated using D', with the coloured squares showing the strength of LD, with black denoting high LD, gray moderate LD and white low LD. The numbers in the block denotes LD calculated using r2. However, the combination specified by block was not found.

## Supplementary Tables

| **Supplementary Table 1. Adjusted odds ratios for CRC patients with specific *HOTAIR* polymorphisms and clinical risk factors.** | | | | | | | | | | | |  |
| --- | --- | --- | --- | --- | --- | --- | --- | --- | --- | --- | --- | --- |
| Characteristics | *HOTAIR* rs7958904 GG vs. GC+CC | |  | *HOTAIR* rs1899663 GG vs. GT+TT | |  | *HOTAIR* rs4759314 AA vs. AG+GG | |  | *HOTAIR* rs920778 TT vs. TC+CC | | |
|  | AOR (95% CI) | AOR (95% CI) |  | AOR (95% CI) | AOR (95% CI) |  | AOR (95% CI) | AOR (95% CI) |  | AOR (95% CI) | AOR (95% CI) | |
| Gender |  |  |  |  |  |  |  |  |  |  |  | |
| Male | 1.000 (reference) | 1.421 (0.937–2.157) |  | 1.000 (reference) | 1.439 (0.943–2.195) |  | 1.000 (reference) | 0.966 (0.523–1.784) |  | 1.000 (reference) | 1.178 (0.777–1.786) | |
| Female | 0.956 (0.661-1.384) | 1.225 (0.810–1.851) |  | 0.945 (0.664–1.346) | 1.236 (0.817–1.871) |  | 0.867 (0.647–1.162) | 0.974 (0.584–1.625) |  | 0.950 (0.658–1.372) | 0.981 (0.652–1.475) | |
| Age |  |  |  |  |  |  |  |  |  |  |  | |
| <62 years | 1.000 (reference) | 1.179 (0.779–1.785) |  | 1.000 (reference) | 1.704 (1.108–2.621) |  | 1.000 (reference) | 0.751 (0.428–1.319) |  | 1.000 (reference) | 0.955 (0.631–1.445) | |
| ≥62 years | 1.098 (0.760-1.587) | 1.719 (1.159–2.551) |  | 1.461 (1.022–2.088) | 1.667 (1.126–2.468) |  | 1.146 (0.848–1.547) | 1.785 (1.008–3.163) |  | 1.080 (0.747–1.561) | 1.441 (0.978–2.121) | |
| HTN |  |  |  |  |  |  |  |  |  |  |  | |
| No | 1.000 (reference) | 1.267 (0.894–1.795) |  | 1.000 (reference) | 1.237 (0.870–1.760) |  | 1.000 (reference) | 1.044 (0.642–1.698) |  | 1.000 (reference) | 1.090 (0.771–1.541) | |
| Yes | 0.655 (0.438-0.979) | 0.999 (0.660–1.512) |  | 0.584 (0.396–0.861) | 1.047 (0.680–1.612) |  | 0.701 (0.511–0.963) | 0.762 (0.405–1.434) |  | 0.700 (0.471–1.041) | 0.842 (0.552–1.285) | |
| DM |  |  |  |  |  |  |  |  |  |  |  | |
| No | 1.000 (reference) | 1.360 (1.010–1.830) |  | 1.000 (reference) | 1.351 (1.000–1.825) |  | 1.000 (reference) | 1.057 (0.694–1.610) |  | 1.000 (reference) | 1.117 (0.831–1.502) | |
| Yes | 1.519 (0.895-2.578) | **2.035 (1.146–3.613)** |  | 1.444 (0.888–2.347) | **2.330 (1.207–4.499)** |  | 1.428 (0.936–2.179) | 1.628 (0.673–3.934) |  | 1.447 (0.862–2.426) | 1.762 (0.984–3.154) | |
| Folate |  |  |  |  |  |  |  |  |  |  |  | |
| >3.08 (nmol/L) | 1.000 (reference) | 1.285 (0.954–1.730) |  | 1.000 (reference) | 1.353 (1.000–1.830) |  | 1.000 (reference) | 1.117 (0.733–1.702) |  | 1.000 (reference) | 1.072 (0.797–1.441) | |
| ≤3.08 (nmol/L) | 2.545 (1.358-4.772) | **3.191 (1.793–5.677)** |  | 2.601 (1.467–4.615) | **3.196 (1.713–5.963)** |  | 2.781 (1.733–4.463) | 1.706 (0.693–4.198) |  | 2.621 (1.392–4.936) | 2.688 (1.521–4.753) | |
| Hcy |  |  |  |  |  |  |  |  |  |  |  | |
| <13.4 (μmol/L) | 1.000 (reference) | 1.386 (1.031–1.864) |  | 1.000 (reference) | 1.313 (0.973–1.773) |  | 1.000 (reference) | 1.068 (0.711–1.607) |  | 1.000 (reference) | 1.050 (0.782–1.410) | |
| ≥13.4 (μmol/L) | 1.718 (0.971-3.040) | 1.899 (1.062–3.398) |  | 1.305 (0.775–2.197) | **2.437 (1.260–4.711)** |  | 1.505 (0.977–2.317) | 1.702 (0.542–5.345) |  | 1.250 (0.707–2.212) | 1.948 (1.089–3.484) | |
| Adjusted odds ratio on the basis of risk factors including age, gender, hypertension, and diabetes mellitus. | | | | | | | | | | | | |

| **Supplementary Table 2. Haplotype analysis for the 4 site HOTAIR polymorphisms in CRC patients and controls** | | | | | |
| --- | --- | --- | --- | --- | --- |
| **Characteristics** | **Overall (2n=1780)** | **Controls  (2n=832)** | **CRC (2n=948)** | **OR (95% CI)** | ***P*^a^** |
| **rs7958904G>C/ rs1899663G>T/ rs4759314A>G/ rs920778T>C** | | | | | |
| G-G-A-T | 0.7032 | 0.6367 | 0.6683 | 1.000 (reference) |  |
| G-G-A-C | 0.0092 | 0.0455 | 0.0285 | 5.336 (1.816-15.680) | 0.001 |
| G-G-G-T | 0.0097 | 0.0121 | 0.0109 | 1.455 (0.406-5.211) | 0.752 |
| G-G-G-C | 0.0039 | 0.0015 | 0.0024 | 0.485 (0.044-5.382) | 0.619 |
| G-T-A-T | 0.0147 | 0.0038 | 0.0090 | 0.323 (0.065-1.616) | 0.174 |
| G-T-A-C | 0.0206 | 0.0008 | 0.0097 | 0.051 (0.003-0.882) | 0.004 |
| G-T-G-T | 0.0023 | 0.0126 | 0.0076 | 5.821 (0.696-48.670) | 0.124 |
| G-T-G-C | 0.0033 | 0.0032 | 0.0033 | 1.940 (0.175-21.530) | 1.000 |
| C-G-A-T | 0.0138 | 0.0038 | 0.0086 | 0.323 (0.065-1.616) | 0.174 |
| C-G-A-C | 0.0172 | 0.0019 | 0.0088 | 0.139 (0.017-1.134) | 0.037 |
| C-G-G-T | 0.0024 | 0.0109 | 0.0066 | 4.851 (0.563-41.790) | 0.217 |
| C-G-G-C | 0.0508 | 0.0407 | 0.0456 | 0.878 (0.462-1.667) | 0.690 |
| C-T-A-T | 0.0114 | 0.0470 | 0.0297 | 4.269 (1.595-11.430) | 0.002 |
| C-T-A-C | 0.1366 | 0.1687 | 0.1542 | 1.362 (0.935-1.983) | 0.107 |
| C-T-G-T | 0.0010 | 0.0073 | 0.0047 | 6.792 (0.349-132.200) | 0.249 |
| C-T-G-C | 0.0000 | 0.0035 | 0.0019 | 4.851 (0.232-101.600) | 0.499 |
| CRC, colorectal cancer; OR, odds ratio; 95% CI, 95% confidence interval; N/A, not applicable; ^a^ Fisher's exact test. | | | | | |

| **Supplementary Table 3. Haplotype analysis for the 3 site HOTAIR polymorphisms in CRC patients and controls** | | | | | |
| --- | --- | --- | --- | --- | --- |
| **Characteristics** | **Overall (2n=1780)** | **Controls  (2n=832)** | **CRC (2n=948)** | **OR (95% CI)** | ***P*^a^** |
| **rs1899663G>T/ rs4759314A>G/ rs920778T>C** | | | | | |
| G-A-T | 0.7145 | 0.6425 | 0.6768 | 1.000 (reference) |  |
| G-A-C | 0.0265 | 0.0481 | 0.0376 | 2.036 (0.975-4.251) | 0.054 |
| G-G-T | 0.0134 | 0.0193 | 0.0162 | 1.461 (0.513-4.155) | 0.475 |
| G-G-C | 0.0558 | 0.0433 | 0.0492 | 0.889 (0.482-1.641) | 0.707 |
| T-A-T | 0.0274 | 0.0479 | 0.0380 | 2.036 (0.975-4.251) | 0.054 |
| T-A-C | 0.1584 | 0.1698 | 0.1644 | 1.180 (0.821-1.697) | 0.370 |
| T-G-T | 0.0032 | 0.0245 | 0.0145 | 11.690 (1.509-90.480) | 0.003 |
| T-G-C | 0.0009 | 0.0047 | 0.0032 | 4.869 (0.233-101.900) | 0.499 |
| **rs7958904G>C/ rs4759314A>G/ rs920778T>C** | | | | | |
| G-A-T | 0.7175 | 0.6398 | 0.6765 | 1.000 (reference) |  |
| G-A-C | 0.0295 | 0.0464 | 0.0384 | 1.803 (0.876-3.710) | 0.105 |
| G-G-T | 0.0122 | 0.0254 | 0.0191 | 2.360 (0.821-6.784) | 0.101 |
| G-G-C | 0.0076 | 0.0047 | 0.0060 | 0.656 (0.109-3.954) | 0.684 |
| C-A-T | 0.0249 | 0.0488 | 0.0374 | 2.262 (1.058-4.835) | 0.031 |
| C-A-C | 0.1547 | 0.1733 | 0.1646 | 1.260 (0.876-1.813) | 0.213 |
| C-G-T | 0.0037 | 0.0203 | 0.0126 | 4.917 (1.068-22.640) | 0.024 |
| C-G-C | 0.0498 | 0.0414 | 0.0455 | 0.937 (0.497-1.764) | 0.839 |
| **rs7958904G>C/ rs1899663G>T/ rs920778T>C** | | | | | |
| G-G-T | 0.7128 | 0.6483 | 0.6790 | 1.000 (reference) |  |
| G-G-C | 0.0119 | 0.0466 | 0.0301 | 4.257 (1.591-11.390) | 0.002 |
| G-T-T | 0.0171 | 0.0170 | 0.0169 | 1.106 (0.396-3.088) | 0.848 |
| G-T-C | 0.0251 | 0.0043 | 0.0138 | 0.194 (0.042-0.891) | 0.019 |
| C-G-T | 0.0156 | 0.0148 | 0.0150 | 1.129 (0.375-3.399) | 0.830 |
| C-G-C | 0.0698 | 0.0434 | 0.0556 | 0.701 (0.391-1.256) | 0.230 |
| C-T-T | 0.0129 | 0.0541 | 0.0345 | 5.031 (1.906-13.280) | 0.0001 |
| C-T-C | 0.1349 | 0.1715 | 0.1550 | 1.399 (0.961-2.038) | 0.079 |
| **rs7958904G>C/ rs1899663G>T/ rs4759314A>G** | | | | | |
| G-G-A | 0.7118 | 0.6816 | 0.6956 | 1.000 (reference) |  |
| G-G-G | 0.0137 | 0.0142 | 0.0140 | 1.069 (0.355-3.218) | 0.905 |
| G-T-A | 0.0359 | 0.0046 | 0.0192 | 0.122 (0.028-0.539) | 0.001 |
| G-T-G | 0.0055 | 0.0158 | 0.0111 | 3.207 (0.661-15.570) | 0.182 |
| C-G-A | 0.0305 | 0.0055 | 0.0171 | 0.212 (0.060-0.750) | 0.008 |
| C-G-G | 0.0542 | 0.0518 | 0.0530 | 0.996 (0.553-1.793) | 0.990 |
| C-T-A | 0.1485 | 0.2165 | 0.1849 | 1.522 (1.070-2.166) | 0.019 |
| C-T-G | 0.0000 | 0.0099 | 0.0050 | 10.08 (0.555-183.200) | 0.063 |
| CRC, colorectal cancer; OR, odds ratio; 95% CI, 95% confidence interval; N/A, not applicable; ^a^ Fisher's exact test. | | | | | |

| **Supplementary Table 4. Haplotype analysis for the 2 site HOTAIR polymorphisms in CRC patients and controls** | | | | | |
| --- | --- | --- | --- | --- | --- |
| **Characteristics** | **Overall (2n=1780)** | **Controls  (2n=832)** | **CRC (2n=948)** | **OR (95% CI)** | ***P*^a^** |
| **rs4759314A>G/ rs920778T>C** | | | | | |
| A-T | 0.7423 | 0.6863 | 0.7126 | 1.000 (reference) |  |
| A-C | 0.1844 | 0.2219 | 0.2042 | 1.297 (0.929-1.809) | 0.126 |
| G-T | 0.0161 | 0.0479 | 0.0329 | 3.124 (1.321-7.386) | 0.007 |
| G-C | 0.0572 | 0.0439 | 0.0503 | 0.832 (0.454-1.525) | 0.551 |
| **rs1899663G>T/ rs920778T>C** | | | | | |
| G-T | 0.7282 | 0.6618 | 0.6930 | 1.000 (reference) |  |
| G-C | 0.0819 | 0.0914 | 0.0868 | 1.220 (0.758-1.966) | 0.412 |
| T-T | 0.0302 | 0.0724 | 0.0525 | 2.524 (1.306-4.876) | 0.005 |
| T-C | 0.1597 | 0.1745 | 0.1677 | 1.214 (0.847-1.739) | 0.291 |
| **rs1899663G>T/ rs4759314A>G** | | | | | |
| G-A | 0.7368 | 0.6898 | 0.7125 | 1.000 (reference) |  |
| G-G | 0.0733 | 0.0634 | 0.0673 | 0.939 (0.553-1.594) | 0.815 |
| T-A | 0.1899 | 0.2184 | 0.2044 | 1.236 (0.887-1.722) | 0.210 |
| T-G | 0.0000 | 0.0284 | 0.0158 | 25.350 (1.500-428.600) | 0.001 |
| **rs7958904G>C/ rs920778T>C** | | | | | |
| G-T | 0.7297 | 0.6651 | 0.6955 | 1.000 (reference) |  |
| G-C | 0.0371 | 0.0511 | 0.0444 | 1.544 (0.795-3.000) | 0.197 |
| C-T | 0.0287 | 0.0691 | 0.0500 | 2.654 (1.345-5.235) | 0.004 |
| C-C | 0.2045 | 0.2147 | 0.2101 | 1.158 (0.834-1.608) | 0.381 |
| **rs7958904G>C/ rs4759314A>G** | | | | | |
| G-A | 0.7464 | 0.6856 | 0.7142 | 1.000 (reference) |  |
| G-G | 0.0204 | 0.0306 | 0.0257 | 1.794 (0.750-4.292) | 0.183 |
| C-A | 0.1803 | 0.2226 | 0.2027 | 1.352 (0.968-1.889) | 0.076 |
| C-G | 0.0529 | 0.0612 | 0.0574 | 1.261 (0.709-2.243) | 0.428 |
| **rs7958904G>C/ rs1899663G>T** | | | | | |
| G-G | 0.7248 | 0.6949 | 0.709 | 1.000 (reference) |  |
| G-T | 0.042 | 0.0213 | 0.0309 | 0.540 (0.243-1.198) | 0.124 |
| C-G | 0.0853 | 0.0582 | 0.0708 | 0.734 (0.436-1.236) | 0.244 |
| C-T | 0.1479 | 0.2255 | 0.1894 | 1.584 (1.117-2.247) | 0.010 |
| CRC, colorectal cancer; OR, odds ratio; 95% CI, 95% confidence interval; N/A, not applicable; ^a^ Fisher's exact test. | | | | | |

| **Supplementary Table 5. Genotype combination analysis for the HOTAIR polymorphisms in CRC patients and controls.** | | | | | | |
| --- | --- | --- | --- | --- | --- | --- |
| Genotype combination | | Controls  (n=416) | CRC (n=474) | AOR (95% CI) | *P* |  |
| SNP 1 | SNP 2 |  |  |  |  |  |
| rs7958904 G>C | rs1899663 G>T |  |  |  |  |  |
| GG | GG | 220 (52.9) | 227 (47.9) | 1.000 (reference) |  |  |
|  | GT | 28 (6.7) | 17 (3.6) | 0.380 (0.184-0.786) | 0.009 |  |
|  | TT | 1 (0.2) | 0 (0) | N/A | 0.994 |  |
| GC | GG | 46 (11.1) | 35 (7.4) | 0.679 (0.414-1.113) | 0.124 |  |
|  | GT | 92 (22.1) | 154 (32.5) | 1.534 (1.110-2.120) | 0.010 |  |
|  | TT | 2 (0.5) | 2 (0.4) | 0.856 (0.117-6.245) | 0.878 |  |
| CC | GG | 5 (1.2) | 2 (0.4) | 0.457 (0.087-2.405) | 0.355 |  |
|  | GT | 12 (2.9) | 15 (3.2) | 0.939 (0.400-2.208) | 0.886 |  |
|  | TT | 10 (2.4) | 22 (4.6) | 2.007 (0.899-4.477) | 0.089 |  |
| rs7958904 G>C | rs4759314 A>G |  |  |  |  |  |
| GG | AA | 236 (56.7) | 226 (47.7) | 1.000 (reference) |  |  |
|  | AG | 13 (3.1) | 14 (3) | 0.974 (0.433-2.191) | 0.949 |  |
|  | GG | 0 (0) | 4 (0.8) | N/A | 0.994 |  |
| GC | AA | 104 (25) | 147 (31) | 1.433 (1.044-1.967) | 0.026 |  |
|  | AG | 35 (8.4) | 43 (9.1) | 1.165 (0.710-1.911) | 0.545 |  |
|  | GG | 1 (0.2) | 1 (0.2) | N/A | 0.994 |  |
| CC | AA | 18 (4.3) | 22 (4.6) | 1.235 (0.623-2.446) | 0.546 |  |
|  | AG | 7 (1.7) | 14 (3) | 1.864 (0.691-5.024) | 0.219 |  |
|  | GG | 2 (0.5) | 3 (0.6) | 1.089 (0.150-7.921) | 0.933 |  |
| rs7958904 G>C | rs920778 T>C |  |  |  |  |  |
| GG | TT | 226 (54.3) | 209 (44.1) | 1.000 (reference) |  |  |
|  | TC | 22 (5.3) | 30 (6.3) | 1.443 (0.797-2.613) | 0.226 |  |
|  | CC | 1 (0.2) | 5 (1.1) | 4.754 (0.546-41.393) | 0.158 |  |
| GC | TT | 12 (2.9) | 42 (8.9) | 3.706 (1.874-7.329) | 0.000 |  |
|  | TC | 122 (29.3) | 144 (30.4) | 1.229 (0.898-1.682) | 0.198 |  |
|  | CC | 6 (1.4) | 5 (1.1) | 0.968 (0.288-3.256) | 0.958 |  |
| CC | TT | 3 (0.7) | 7 (1.5) | 1.910 (0.445-8.203) | 0.384 |  |
|  | TC | 5 (1.2) | 6 (1.3) | 1.656 (0.486-5.636) | 0.420 |  |
|  | CC | 19 (4.6) | 26 (5.5) | 1.366 (0.703-2.652) | 0.358 |  |
| rs1899663 G>T | rs4759314 A>G |  |  |  |  |  |
| GG | AA | 226 (54.3) | 226 (47.7) | 1.000 (reference) |  |  |
|  | AG | 42 (10.1) | 34 (7.2) | 0.758 (0.459-1.249) | 0.277 |  |
|  | GG | 3 (0.7) | 4 (0.8) | 1.087 (0.213-5.551) | 0.920 |  |
| GT | AA | 119 (28.6) | 148 (31.2) | 1.189 (0.872-1.621) | 0.273 |  |
|  | AG | 13 (3.1) | 34 (7.2) | 2.187 (1.094-4.372) | 0.027 |  |
|  | GG | 0 (0) | 4 (0.8) | N/A | N/A |  |
| TT | AA | 13 (3.1) | 21 (4.4) | 1.517 (0.715-3.219) | 0.278 |  |
|  | AG | 0 (0) | 3 (0.6) | N/A | 0.993 |  |
|  | GG | 0 (0) | 0 (0) | N/A | N/A |  |
| rs1899663 G>T | rs920778 T>C |  |  |  |  |  |
| GG | TT | 223 (53.6) | 203 (42.8) | 1.000 (reference) |  |  |
|  | TC | 43 (10.3) | 54 (11.4) | 1.308 (0.832-2.057) | 0.245 |  |
|  | CC | 5 (1.2) | 7 (1.5) | 1.620 (0.500-5.247) | 0.421 |  |
| GT | TT | 17 (4.1) | 52 (11) | 2.841 (1.561-5.168) | 0.001 |  |
|  | TC | 102 (24.5) | 122 (25.7) | 1.251 (0.898-1.743) | 0.185 |  |
|  | CC | 13 (3.1) | 12 (2.5) | 0.866 (0.368-2.039) | 0.743 |  |
| TT | TT | 1 (0.2) | 3 (0.6) | 3.077 (0.314-30.149) | 0.335 |  |
|  | TC | 4 (1) | 4 (0.8) | 1.241 (0.302-5.103) | 0.764 |  |
|  | CC | 8 (1.9) | 17 (3.6) | 2.114 (0.853-5.238) | 0.106 |  |
| rs4759314 A>G | rs920778 T>C |  |  |  |  |  |
| AA | TT | 230 (55.3) | 228 (48.1) | 1.000 (reference) |  |  |
|  | TC | 112 (26.9) | 141 (29.7) | 1.265 (0.924-1.734) | 0.143 |  |
|  | CC | 16 (3.8) | 26 (5.5) | 1.497 (0.764-2.933) | 0.239 |  |
| AG | TT | 11 (2.6) | 27 (5.7) | 2.200 (1.044-4.638) | 0.038 |  |
|  | TC | 37 (8.9) | 36 (7.6) | 0.896 (0.539-1.490) | 0.672 |  |
|  | CC | 7 (1.7) | 8 (1.7) | 0.947 (0.305-2.940) | 0.925 |  |
| GG | TT | 0 (0) | 3 (0.6) | N/A | N/A |  |
|  | TC | 0 (0) | 3 (0.6) | N/A | 0.994 |  |
|  | CC | 3 (0.7) | 2 (0.4) | 0.744 (0.121-4.579) | 0.750 |  |
| CRC colorectal cancer; AOR, adjusted odds ratio; 95% CI, 95% confidence interval; N/A, not applicable. ^a^ Adjusted by age | | | | | | |

| **Supplementary Table 6. Genotype combination analysis for the HOTAIR polymorphisms in CRC subtypes and controls.** | | | | | | | | |
| --- | --- | --- | --- | --- | --- | --- | --- | --- |
| Genotype combination | | Controls  (n=416) | Colon (n=272) | AOR (95% CI) | *P* | Rectum (n=189) | AOR (95% CI) | *P* |
| SNP 1 | SNP 2 |  |  |  |  |  |  |  |
| rs7958904 G>C | rs1899663 G>T |  |  |  |  |  |  |  |
| GG | GG | 220 (52.9) | 140 (51.5) | 1.000 (reference) |  | 85 (45) | 1.000 (reference) |  |
|  | GT | 28 (6.7) | 11 (4) | 0.445 (0.195-1.014) | 0.054 | 4 (2.1) | 0.350 (0.119-1.031) | 0.057 |
|  | TT | 1 (0.2) | 0 (0) | N/A | 0.995 | 0 (0) | N/A | 0.995 |
| GC | GG | 46 (11.1) | 21 (7.7) | 0.668 (0.372-1.198) | 0.176 | 14 (7.4) | 0.715 (0.366-1.398) | 0.327 |
|  | GT | 92 (22.1) | 81 (29.8) | 1.404 (0.969-2.035) | 0.073 | 68 (36) | 1.833 (1.227-2.740) | 0.003 |
|  | TT | 2 (0.5) | 0 (0) | N/A | 0.995 | 2 (1.1) | 2.309 (0.309-17.256) | 0.415 |
| CC | GG | 5 (1.2) | 0 (0) | N/A | 0.995 | 2 (1.1) | 1.144 (0.215-6.096) | 0.875 |
|  | GT | 12 (2.9) | 8 (2.9) | 1.187 (0.460-3.063) | 0.724 | 4 (2.1) | 0.803 (0.248-2.602) | 0.715 |
|  | TT | 10 (2.4) | 11 (4) | 1.924 (0.759-4.874) | 0.168 | 10 (5.3) | 2.241 (0.859-5.849) | 0.099 |
| rs7958904 G>C | rs4759314 A>G |  |  |  |  |  |  |  |
| GG | AA | 236 (56.7) | 142 (52.2) | 1.000 (reference) |  | 83 (43.9) | 1.000 (reference) |  |
|  | AG | 13 (3.1) | 6 (2.2) | 0.882 (0.341-2.284) | 0.796 | 6 (3.2) | 1.539 (0.589-4.018) | 0.379 |
|  | GG | 0 (0) | 3 (1.1) | N/A | 0.993 | 0 (0) | N/A | N/A |
| GC | AA | 104 (25) | 76 (27.9) | 1.296 (0.899-1.871) | 0.165 | 6 (3.2) | 1.774 (1.192-2.641) | 0.005 |
|  | AG | 35 (8.4) | 25 (9.2) | 1.080 (0.608-1.917) | 0.793 | 18 (9.5) | 1.304 (0.683-2.489) | 0.421 |
|  | GG | 1 (0.2) | 1 (0.4) | N/A | 0.995 | 0 (0) | N/A | 0.996 |
| CC | AA | 18 (4.3) | 11 (4) | 1.157 (0.508-2.635) | 0.729 | 10 (5.3) | 1.427 (0.607-3.353) | 0.415 |
|  | AG | 7 (1.7) | 8 (2.9) | 2.496 (0.838-7.435) | 0.100 | 4 (2.1) | 1.584 (0.442-5.670) | 0.480 |
|  | GG | 2 (0.5) | 0 (0) | N/A | 0.995 | 2 (1.1) | 3.213 (0.438-23.586) | 0.251 |
| rs7958904 G>C | rs920778 T>C |  |  |  |  |  |  |  |
| GG | TT | 226 (54.3) | 129 (47.4) | 1.000 (reference) |  | 76 (40.2) | 1.000 (reference) |  |
|  | TC | 22 (5.3) | 19 (7) | 1.550 (0.794-3.027) | 0.199 | 11 (5.8) | 1.252 (0.565-2.774) | 0.580 |
|  | CC | 1 (0.2) | 3 (1.1) | 5.249 (0.53-51.983) | 0.156 | 2 (1.1) | 5.732 (0.494-66.549) | 0.163 |
| GC | TT | 12 (2.9) | 18 (6.6) | 2.644 (1.206-5.797) | 0.015 | 23 (12.2) | 5.591 (2.620-11.933) | <0.0001 |
|  | TC | 122 (29.3) | 80 (29.4) | 1.177 (0.818-1.693) | 0.380 | 61 (32.3) | 1.359 (0.905-2.040) | 0.140 |
|  | CC | 6 (1.4) | 4 (1.5) | 1.589 (0.469-5.385) | 0.457 | 0 (0) | 0.570 (0.066-4.904) | 0.609 |
| CC | TT | 3 (0.7) | 2 (0.7) | 1.512 (0.237-9.654) | 0.662 | 3 (1.6) | 2.780 (0.540-14.311) | 0.221 |
|  | TC | 5 (1.2) | 4 (1.5) | 2.511 (0.681-9.264) | 0.167 | 1 (0.5) | 1.109 (0.201-6.119) | 0.905 |
|  | CC | 19 (4.6) | 13 (4.8) | 1.315 (0.590-2.931) | 0.504 | 12 (6.3) | 1.511 (0.664-3.438) | 0.325 |
| rs1899663 G>T | rs4759314 A>G |  |  |  |  |  |  |  |
| GG | AA | 226 (54.3) | 141 (51.8) | 1.000 (reference) |  | 84 (44.4) | 1.000 (reference) |  |
|  | AG | 42 (10.1) | 18 (6.6) | 0.687 (0.378-1.251) | 0.220 | 15 (7.9) | 0.942 (0.494-1.797) | 0.856 |
|  | GG | 3 (0.7) | 2 (0.7) | 0.604 (0.061-6.028) | 0.668 | 2 (1.1) | 2.170 (0.348-13.513) | 0.407 |
| GT | AA | 119 (28.6) | 78 (28.7) | 1.085 (0.757-1.555) | 0.659 | 65 (34.4) | 1.441 (0.973-2.135) | 0.068 |
|  | AG | 13 (3.1) | 20 (7.4) | 2.517 (1.194-5.305) | 0.015 | 11 (5.8) | 2.125 (0.891-5.066) | 0.089 |
|  | GG | 0 (0) | 2 (0.7) | N/A | N/A | 0 (0) | N/A | N/A |
| TT | AA | 13 (3.1) | 10 (3.7) | 1.354 (0.550-3.333) | 0.510 | 10 (5.3) | 1.830 (0.736-4.549) | 0.193 |
|  | AG | 0 (0) | 1 (0.4) | N/A | 0.993 | 2 (1.1) | N/A | 0.993 |
|  | GG | 0 (0) | 0 (0) | N/A | N/A | 0 (0) | N/A | N/A |
| rs1899663 G>T | rs920778 T>C |  |  |  |  |  |  |  |
| GG | TT | 223 (53.6) | 124 (45.6) | 1.000 (reference) |  | 77 (40.7) | 1.000 (reference) |  |
|  | TC | 43 (10.3) | 34 (12.5) | 1.372 (0.821-2.294) | 0.228 | 20 (10.6) | 1.231 (0.671-2.257) | 0.502 |
|  | CC | 5 (1.2) | 3 (1.1) | 1.324 (0.304-5.770) | 0.709 | 4 (2.1) | 2.524 (0.651-9.795) | 0.181 |
| GT | TT | 17 (4.1) | 25 (9.2) | 2.427 (1.230-4.788) | 0.011 | 22 (11.6) | 3.704 (1.863-7.367) | 0.000 |
|  | TC | 102 (24.5) | 66 (24.3) | 1.209 (0.823-1.777) | 0.333 | 52 (27.5) | 1.394 (0.912-2.131) | 0.125 |
|  | CC | 13 (3.1) | 9 (3.3) | 1.321 (0.541-3.226) | 0.541 | 2 (1.1) | 0.428 (0.094-1.954) | 0.273 |
| TT | TT | 1 (0.2) | 3 (1.1) | N/A | 0.995 | 3 (1.6) | 8.195 (0.817-82.204) | 0.074 |
|  | TC | 4 (1) | 8 (2.9) | 1.656 (0.353-7.757) | 0.522 | 1 (0.5) | 0.641 (0.069-5.949) | 0.696 |
|  | CC | 8 (1.9) | 0 (0) | 2.015 (0.692-5.864) | 0.199 | 8 (4.2) | 2.417 (0.829-7.046) | 0.106 |
| rs4759314 A>G | rs920778 T>C |  |  |  |  |  |  |  |
| AA | TT | 230 (55.3) | 138 (50.7) | 1.000 (reference) |  | 88 (46.6) | 1.000 (reference) |  |
|  | TC | 112 (26.9) | 77 (28.3) | 1.232 (0.855-1.775) | 0.264 | 61 (32.3) | 1.363 (0.914-2.034) | 0.129 |
|  | CC | 16 (3.8) | 14 (5.1) | 1.623 (0.757-3.483) | 0.214 | 10 (5.3) | 1.577 (0.686-3.625) | 0.284 |
| AG | TT | 11 (2.6) | 10 (3.7) | 1.577 (0.648-3.840) | 0.315 | 14 (7.4) | 3.585 (1.579-8.141) | 0.002 |
|  | TC | 37 (8.9) | 23 (8.5) | 1.024 (0.579-1.811) | 0.935 | 12 (6.3) | 0.777 (0.378-1.597) | 0.493 |
|  | CC | 7 (1.7) | 6 (2.2) | 1.492 (0.444-5.015) | 0.518 | 2 (1.1) | 0.359 (0.043-2.992) | 0.343 |
| GG | TT | 0 (0) | 1 (0.4) | N/A | N/A | 0 (0) | N/A | N/A |
|  | TC | 0 (0) | 3 (1.1) | N/A | 0.993 | 0 (0) | N/A | 0.993 |
|  | CC | 3 (0.7) | 0 (0) | N/A | 0.994 | 2 (1.1) | 1.859 (0.301-11.487) | 0.504 |
| CRC colorectal cancer; AOR, adjusted odds ratio; 95% CI, 95% confidence interval; N/A, not applicable. ^a^ Adjusted by age | | | | | | | | |

| **Supplementary Table 7. Statistical powers of genetic associations in the present case-control study** | | | |
| --- | --- | --- | --- |
| **Characteristics** | **Association** | **AOR (95% CI)** | **Statistical power (%)** |
| *HOTAIR* rs7958904 GC | Total CRC patients | 1.352 (1.014–1.801) | 62.76% |
| *HOTAIR rs7958904* Dominant model | Total CRC patients | 1.351 (1.027–1.777) | 69.57% |
| *HOTAIR* rs7958904 GC | Rectum patients | 1.559 (1.076–2.258) | 78.56% |
| *HOTAIR rs7958904* Dominant model | Rectum patients | 1.547 (1.085–2.205) | 81.74% |
| *HOTAIR* rs1899663 GT | Total CRC patients | 1.338 (1.004–1.784) | 72.66% |
| *HOTAIR* rs1899663 Dominant model | Total CRC patients | 1.378 (1.043–1.822) | 81.31% |
| *HOTAIR* rs1899663 Dominant model | Rectum patients | 1.481 (1.035–2.12) | 77.13% |
| *Note:* CRC, colorectal cancer; AOR, adjusted odd ratio. | | |  |
